# Supplementary material for: Economic evaluation of insulin glargine compared with human insulin for youth with type 1 diabetes in Tanzania and Bangladesh
Source: PLoS One. 2026 Jan 2;21(1):e0333652. doi: 10.1371/journal.pone.0333652 (PMC12758729; doi:10.1371/journal.pone.0333652)
Supplement: S1 Table — (DOCX) [file pone.0333652.s001.docx]

**S1 Table. Targeted sensitivity analysis results**

|  |  |  |  |  |  |  |
| --- | --- | --- | --- | --- | --- | --- |
| **Assumption / Input** | **Country** | **Base case** | **Sensitivity input** | **Cost per patient per year (USD)** | **Total disutility** | **ICER** |
| **Base case outputs** | Tanzania | For NPH and Reg in Vials Glargine in pens | n/a | NPH: $758 IGlar: $1,140 | NPH: 0.0941 IGlar: 0.1029 | 43,508 |
|  | Bangladesh | For NPH, Reg, and Glargine in cartridges with reusable pens | n/a | NPH: $712 IGlar: $770 | NPH: 0.0941 IGlar: 0.1029 | 6,663 |
| **Alcohol swabs** | Tanzania | Used for each SMBG and injection | Not used at all | NPH: $730 IGlar: $1,107 | NPH: 0.0941 IGlar: 0.1029 | 42,990 |
|  | Bangladesh |  |  | NPH: $691 IGlar: $752 | NPH: 0.0941 IGlar: 0.1029 | 6,951 |
| **Syringe / needle reuse** | Tanzania | Used 4 times  (Klatman and Ogle, 2020) | Not re-used | NPH: $860 IGlar: $1,354 | NPH: 0.0941 IGlar: 0.1029 | 56,396 |
|  | Bangladesh |  |  | NPH: $864 IGlar: $892 | NPH: 0.0941 IGlar: 0.1029 | 3,196 |
| **Total daily insulin dose** | Tanzania | Endline HumAn-1 values by arm:  SOC: 1.31 IUs/kg/day IGlar: 1.15 IUs/kg/day (signif. diff) | Baseline HumAn-1 values for overall sample: 1.04 IUs/kg/day | NPH: $723 IGlar: $1,089 | NPH: 0.0941 IGlar: 0.1029 | 41,709 |
|  | Bangladesh |  |  | NPH: $651 IGlar: $734 | NPH: 0.0941 IGlar: 0.1029 | 9,499 |
| **Daily self-monitoring of blood glucose** | Tanzania | 3 times per day (as per clinical guidance) | 3 times per week (more in line with actual practice) | NPH: $409 IGlar: $791 | NPH: 0.0941 IGlar: 0.1029 | 43,508 |
|  | Bangladesh |  |  | NPH: $481 IGlar: $539 | NPH: 0.0941 IGlar: 0.1029 | 6,663 |
| **Low-end costs** | Tanzania | Manufacturer's suggested retail prices, or most commonly observed retail prices for all items | Lowest observed retail prices for all items | NPH: $594 IGlar: $915 | NPH: 0.0941 IGlar: 0.1029 | 36,687 |
|  | Bangladesh |  |  | NPH: $540 IGlar: $601 | NPH: 0.0941 IGlar: 0.1029 | 6,986 |
| **High-end costs** | Tanzania | Manufacturer's suggested retail prices, or most commonly observed retail prices for all items | Highest observed retail prices for all items | NPH: $1,025 IGlar: $1,478 | NPH: 0.0941 IGlar: 0.1029 | 51,725 |
|  | Bangladesh |  |  | NPH: $1,168 IGlar: $1,130 | NPH: 0.0941 IGlar: 0.1029 | -4,337 |
| **50% of retail prices for insulin** | Tanzania | Manufacturer's suggested retail prices, or most commonly observed retail prices for all items | 50% of MSRP or retail prices for insulin (to simulate procurement pricing) | NPH: $673 IGlar: $874 | NPH: 0.0941 IGlar: 0.1029 | 22,945 |
|  | Bangladesh |  |  | NPH: $564 IGlar: $582 | NPH: 0.0941 IGlar: 0.1029 | 2,027 |
| **Reduced nocturnal hypoglycemic events per year in both arms** | Tanzania | Endline HumAn-1 values by arm:  SOC: 109.2 events per year IGlar: 83.2 events per year | Assuming that 40% of events are symptomatic (Divilly et al 2024; Pazos-Couselo et al 2015) SOC: 33.3 events per year IGlar: 43.7 events per year | NPH: $758 IGlar: $1,140 | NPH: 0.0697 IGlar: 0.0762 | 58,746 |
|  | Bangladesh |  |  | NPH: $712 IGlar: $770 | NPH: 0.0697 IGlar: 0.0762 | 8,996 |

Note: ICERs that are below 3x GDP per capita threshold for cost-effectiveness are shown in bold underlining.
